# The expression of signal molecules in buccal epithelium: new abilities of molecular personalized diagnosis of human diseases

**DOI:** 10.1186/1878-5085-5-S1-A55

**Published:** 2014-02-11

**Authors:** Michail A Paltsev, Igor M Kvetnoy, Victoria O Polyakova, Kirill L Kozlov, Igor B Oleksyuk

**Affiliations:** 1 National Research Centre «Kurchatov Institute»; Ott Institute of Obstetrics and Gynecology RAMS; Moscow, Saint-Petersburg, Russian Federation

## Scientific objectives

The actual problem of molecular medicine is the search of cells, receipt of which is may be possible by non-invasive way, which opens up the possibility of life-time diagnosis of social important diseases. It seems, that one of the most promising objects for this is the buccal epithelium (BE), which cells express a large number of signaling molecules involved in local and systemic regulation of the homeostasis as well as in pathogenesis of many pathologies.

## The aim of research

To study of the expression of some signaling molecules in human BE, which could be useful for personalized diagnosis of human diseases.

## Technological approaches

The BE samples were taken from patients with breast cancer without metastases (BC), with Alzheimer's disease (AD) and with coronary atherosclerosis (AT) and also from healthy volunteers (control, CO). BE cytological smears were prepared by liquid-based cytology. For immunocytochemical studies we used primary monoclonal antibody to CD64, CD90, Oct2 (for BC), to tau protein and β-APP (for BA), to connexin Cx37 and Cx40 (for AT) and secondary antibodies - biotinylated anti-mouse immunoglobulins (all Dako). Determination of the average area of marker expression was performed by morphometric method using the system of computer analysis of microscopic images (Nikon Eclipse400) as well as the licensed program Morphology 5.0 (Videotest) followed by statistical analysis of the results.

## The results interpretation

In CO group all the studied markers were verified and determined their average area of expression. In BC group has been shown the reduction of expression of markers of immune cells SD64 on 92%, CD90 on 46%, Oct2 on 28% compared to CO. In AD group the synthesis of tau protein and β-APP was increased on 45% and on 37% respectively compared to CO. In AT group expression of Cx37 and Cx40 was decreased on 61% and 39% respectively compared with CO. The correlations between the levels of expression of biomarkers and stages of diseases were established for all pathologies studied.

## Outlook and expert recommendations

The results indicate that BE could be considered as an informative object for personalized molecular diagnosis of human diseases.

